# Implications of a clinical medication review and a pharmaceutical care plan of polypharmacy patients with a cardiovascular disorder

**DOI:** 10.1007/s11096-016-0281-x

**Published:** 2016-04-06

**Authors:** Marlies M. E. Geurts, Roy E. Stewart, Jacobus R. B. J. Brouwers, Pieter A. de Graeff, Johan J. de Gier

**Affiliations:** Department Pharmacotherapy and Pharmaceutical Care, University of Groningen, Antonius Deusinglaan 1, 9713 AV Groningen, The Netherlands; Department Community and Occupational Health, University Medical Center Groningen, Groningen, The Netherlands; Department Clinical Pharmacy and Pharmacology, University Medical Center Groningen, Groningen, The Netherlands

**Keywords:** Community pharmacy, Netherlands, Pharmaceutical care, Pharmacist consultation, Pharmacy practice, Polypharmacy, Safety

## Abstract

*Background* A clinical medication review, including patient involvement, is expected to improve pharmaceutical care. *Objective* To determine whether a clinical medication review followed by a pharmaceutical care plan decreases the number of potential drug-related problems (DRPs) and pharmaceutical care issues (PCIs) and leads to a positive effect on relevant clinical and laboratory parameters for elderly cardiovascular patients with multiple drug use. *Setting* Randomized controlled trial in eight primary care settings in the Netherlands. *Method* Elderly polypharmacy patients with a cardiovascular disorder were randomized into two groups. Intervention patients received a clinical medication review, followed by a pharmaceutical care plan developed in cooperation between these patients’ pharmacists and general practitioners (GPs), and agreed to by the patients. Control patients received care as usual. Patient data were collected at the start of the study (t = 0) and after 1-year follow-up (t = 1). *Main outcome measure* Decrease in potential DRPs and pharmaceutical PCIs, improvement of clinical and laboratory parameters. *Results* 512 patients were included. An average of 2.2 potential DRPs and pharmaceutical PCIs were defined per patient in the intervention group. After 1-year follow-up, 47.2 % of potential DRPs and PCIs were resolved. In total, 156 care interventions were proposed (0.9/patient), 108 of which were implemented after 1 year (69.2 %). For control-group patients, a total of 47 proposed care interventions were documented for 255 patients (0.2/patient); after 1 year, 43 had been implemented (91.5 %). The study intervention (*p* < 0.001) and the number of medicines used (*p* = 0.030) had a significant effect on the number of interventions proposed. Small biochemical changes in cardiovascular risk factors did occur, but the differences were small and not considered clinically relevant. *Conclusion* The integrated use of a clinical medication review with a pharmaceutical care plan in a primary care setting supports the detection of and decrease in DRPs and pharmaceutical PCIs in almost half of the patients. Its benefit in terms of control of cardiovascular risk factors and safety parameters was relatively low. Risk stratification might be necessary to decide which patients might benefit most from this type of intervention.

## Impacts of findings on practice statements

- Risk stratification is important in order to define patients who benefit most from the intervention;
- Besides the appropriate knowledge, sufficient time and reimbursement are important to implement clinical pharmacy services in daily practice.

## Introduction

Appropriate prescribing in elderly people needs more attention [1]. The number of medications taken may affect quality of prescribing and adherence in older persons [2, 3]. A regular medication review (MR) has the potential to improve pharmaceutical care in patients [4, 5]. This type of review is defined as “a structured, critical examination of a patient’s medicines with the objective of reaching an agreement with the patient about treatment, optimizing the impact of medicines, minimizing the number of medication-related problems and reducing waste” [6]. Only a clinical medication review (CMR), including pharmacist, general practitioner (GP), and patient [6], can be expected to improve pharmaceutical care [4]. Patient involvement is important both for the identification of drug-related problems (DRPs) [7] and for the long-term success of the intervention performed [4].

Various MR methods have been described. Clinical pharmacists are able to review care-home patients’ medication and make recommendations to GPs [8]. A decrease in DRPs was shown after a pharmacist-conducted MR of elderly patients receiving medicines via automated dispensing machines [9]. In a hospital setting, it was concluded that structured pharmaceutical care, according to a protocol, leads to more changes in drug therapy compared to care as usual [10]. A CMR intervention, including pharmacists, GPs, and patients, has demonstrated that such an intervention may prevent medication-related hospital admissions but without any statistically significant effect on the number of adverse drug events, quality of life, or survival [11, 12]. Assessment of a patients’ pharmacotherapy includes checking whether all indications are treated appropriately, whether the medication treatment is effective and safe, and whether a patient has adhered to the proposed therapy. Potential problems concerning pharmacotherapy can be defined as a potential DRP, based on the concept of Cipolle et al. [13], or on the basis of a pharmaceutical care issue (CI) [11]. Literature defines different sub-groups of patients with known non-adherence and/or medication problems [14, 15]. Methods for enhancing medication safety in older persons may be directed towards aspects of specific types of drugs, such as anticholinergic drug burden, under-prescribing, or the use of the Medication Appropriateness Index, Beers criteria, or the STOPP and START criteria [16–20]. Elderly patients with a cardiovascular disorder use multiple medicines that require regular monitoring using relevant clinical and laboratory parameters related to cardiovascular risk assessment (blood pressure and cholesterol levels) and safety (renal function and potassium). This patient population could benefit from a CMR [15] with adequate follow-up and was therefore chosen as our study population. Compared to other studies, we combined a CMR with a web-based pharmaceutical care plan (W-PCP) to facilitate integrated care and to systematically structure joint use of patients’ medical and pharmaceutical records and to document the integrated information and interventions for follow-up. Moreover, we performed this study in daily practice, and not in a research setting, so that healthcare providers could implement the intervention as realistic as possible in their daily routine.

## Aim of the study

To determine whether a CMR followed by a pharmaceutical care plan (PCP) decreases potential DRPs and PCIs, along with a positive effect on cardiovascular risk factors and safety parameters for elderly polypharmacy patients with a cardiovascular disorder.

## Ethics approval

An independent Ethics Committee (RTPO/Leeuwarden, the Netherlands) reviewed the study protocol. The protocol was graded as a clinical intervention study with no risk for patients. To guarantee patient privacy, patient data were made anonymous before the database was provided to the researchers.

## Method

A randomized controlled trial was performed in the primary care setting of the Netherlands. Community pharmacists (n = 500; 25 % of all pharmacies in the Netherlands) were invited by letter to participate. Pharmacies were randomly selected in an area defined by the sponsor of the study. After consenting, the pharmacists subsequently contacted GPs and asked for their participation. Good cooperation between pharmacists and GPs, and the willingness to share patient data were prerequisites. Participating pharmacies and GP practices were connected to a newly developed W-PCP application [21]. This web-based application uploaded all patient data from pharmacy and GP computer systems in order to combine information about diagnoses, medicines prescribed, and clinical and laboratory parameters in one patient file, accessible to both patient’s pharmacist and GP. Patients were included based on inclusion criteria from screening pharmacy records:

- aged ≥60 years;
- elderly patients with polypharmacy (five or more medicines for chronic use);
- of which at least one medicine for a cardiovascular disorder (ATC class C [22]).

Patients who did not speak Dutch language or who were mentally impaired were excluded.

Patient inclusion transpired between August 2009 and June 2010. After informed consent, patients were randomized into an intervention or a control group. Randomization occurred based on unique patient identification numbers (IDs) in the pharmacy computer system (odd number: intervention group; even number: control group). Intervention patients received an invitation to consult their pharmacist for a CMR. The PCP was subsequently developed in cooperation between patient’s pharmacist and GP, and agreed to by the patient. Each evaluation of the PCP for intervention patients consisted of three components: (1) potential DRPs and PCIs, (2) proposed care interventions to reach treatment goals, and (3) implemented care interventions. Patients from the control group received care as usual and were not treated differently than before. All patients were followed-up for 1 year. Total study period per site was 18 months, with consultations and MRs performed during the first 6 months. The last data collection finished in December 2011.

### Support

A learning module of the W-PCP application was provided by the researchers to all participating pharmacists and GPs. During the study period technical assistance was available. All participating pharmacists received a 1-day training course on communication skills with GPs and patients. Additional written information about performing a CMR was provided. During the study period, researchers visited study sites regularly in order to monitor the time schedule of the study and provide assistance.

### Data collection

Patient data were uploaded regularly, depending upon patients’ consultations, and collected in the W-PCP application. Two measurements were performed, one at the beginning of the study (t = 0) and one after 1-year follow-up (t = 1). Patient data consisted of general patient information (age, gender), episodes (ICPC-coded [23]), medicines dispensed (ATC-coded [22]), and clinical and laboratory parameters. Patient data was provided to the researchers in a database (Microsoft Access 2010).

Primary outcome of this study was a decrease in potential DRPs and PCIs, expressed as a percentage of resolved DRPs and PCIs. Secondary outcome was the differences in clinical and laboratory parameters. PCPs consisted of “free text” entered by the healthcare providers. Two researchers (MG, author, and EM, not an author) coded all individual care plans independently. All codes were compared and inconsistencies discussed until agreement was reached. For control-group patients, information on care interventions was collected retrospectively.

### Sample size calculation and analysis

Two sample size calculations were performed. Our aim was to demonstrate a 25 % decrease in potential DRPs and PCIs. Based on a paired means power analysis using simulation and Wilcoxon signed-rank test using alpha = 0.05 and a power of 0.80, we needed 13 patients per pharmacy in the intervention group. The second aim was to demonstrate a 10 % improvement in clinical and laboratory parameters. Based on a two independent proportions power analysis using alpha = 0.05 and a power of 0.80, we needed 400 patients for each group. Our aim was to recruit patients from 10 to 12 study sites. Based on our second sample size calculation, and considering dropouts as a consequence of losses to follow-up, our aim was to include 100 patients per study site.

Statistical analyses were performed using SPSS 21, Mplus 7.1, and SAS 9.3. Differences in patient characteristics were calculated using 1-way ANOVA and Pearson Chi square test. Multilevel analysis was used to analyze the nesting structure. Effects of the study intervention on number of care interventions performed were analyzed using a two level Multilevel analysis with Poisson regression analysis, where patients (level 1) were nested within GPs (level 2). As model information, we used the Akaike information criteria (AIC) and the Bayesian information citeria (BIC) to compare the relative goodness-of-fit of the presented models. Effect of the study intervention on clinical endpoints was analyzed using a three level Multilevel analysis, including measurements (t = 0 and t = 1) (level 1) nested within patients (level 2) and patients nested within GPs (level 3). Results were considered statistically significant at a significance level *p* < 0.05.

## Results

**Fig. 1:**
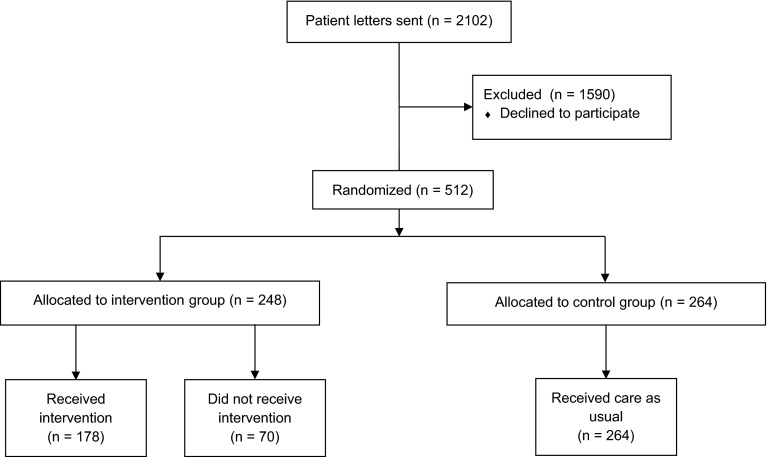
Patient recruitment and randomization

Eight study sites recruited 512 patients: 248 in the intervention group, 264 in the control group. Pharmacists and GPs did not manage to perform CMRs for all intervention patients during the study period due to time limitations. Therefore, 70 patients, originally randomized into the intervention group, were analyzed as a separate group since they did not receive any part of the intervention (Fig. 1). Patient characteristics were comparable between groups (Table 1).

**Table 1:** Patient characteristics (n = 512) at time of inclusion (t = 0)

	Intervention patients with intervention n = 178 [mean (SD)]	Intervention patients without intervention n = 70 [mean (SD)]	Control n = 264 [mean (SD)]	*p* value
Age (years)	72.5 (7.735)	71.8 (8.372)	73.1 (7.797)	0.433^c^
Gender, male (%)	46.1	52.9	47.3	0.622^d^
# Medicines^a^	8.3 (2.721)	8.0 (3.277)	7.9 (2.926)	0.591^c^
# Episodes^b^	14.6 (8.210)	14.3 (6.475)	14.8 (8.683)	0.891^c^

SD, standard deviation; #, number

^a^ATC-coded [22]

^b^ICPC-coded [23]

^c^One-way ANOVA

^d^Pearson Chi square test

**Fig. 2:**
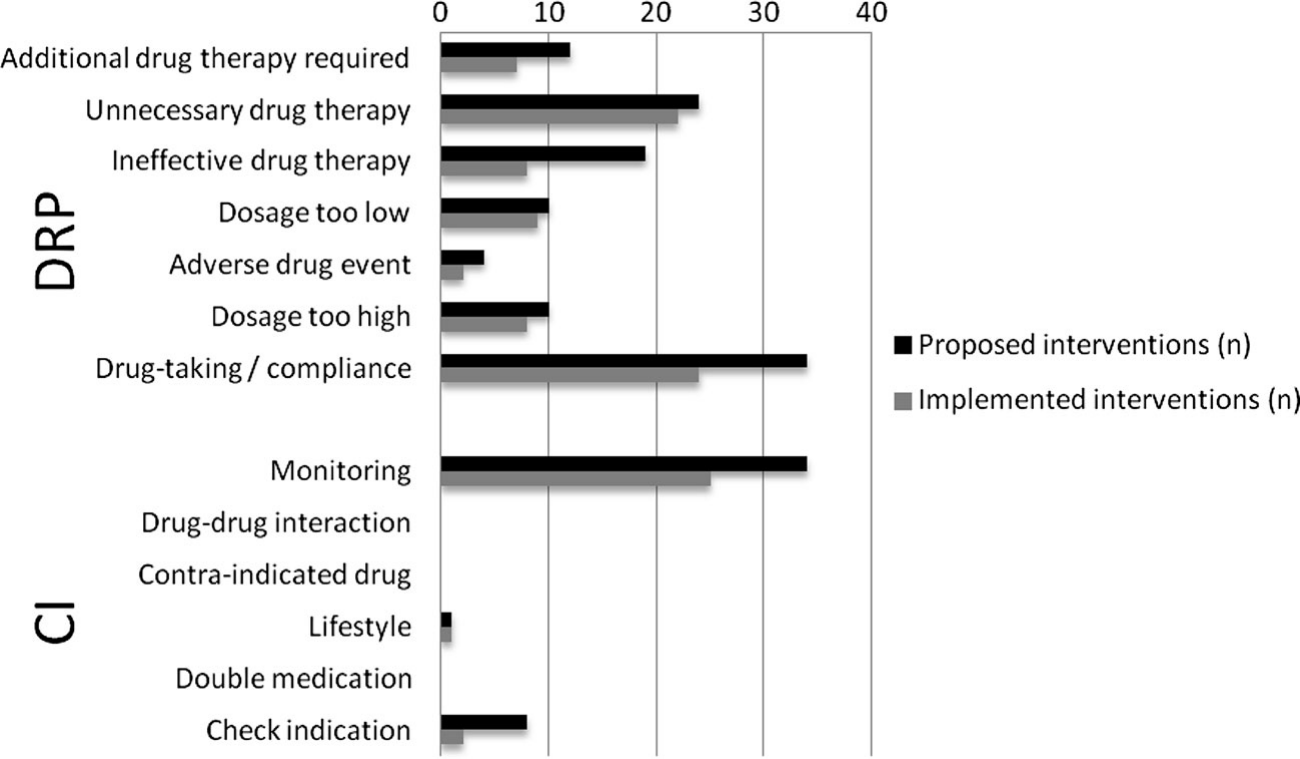
Number of proposed and implemented interventions based on DRPs/PCIs retrieved from the pharmaceutical care plan (n = 178). *DRP* drug related problem, *CI* care issue

In total, 394 potentially harmful DRPs and PCIs were defined for 178 intervention patients (2.2/patient). After 1-year follow-up, 186 potential DRPs and PCIs (47.2 %) were resolved; 208 DRPs and PCIs (1.2/patient) were not resolved or with unknown outcome from the available data. During the study period, 156 care interventions were proposed (0.9/patient) (range 0–5 per patient) of which 108 were implemented after 1 year (69.2 %). Figure 2 shows the number of proposed and implemented care interventions per category. Most proposed care interventions were related to drug-taking/adherence, monitoring (e.g., additional clinical values), and unnecessary drug therapy. Categories with the most implemented interventions: unnecessary drug therapy (e.g., stop medicine) (91.7 %), dosage too low (e.g., increase dosage) (90.0 %), and dosage too high (e.g., decrease dosage) (80.0 %).

In the control group, a total of 47 proposed care interventions were documented for 255 patients (0.2/patient) (range 0–4 per patient). Information was missing for 9 patients. Of the 47 proposed care interventions, 43 were implemented after 1 year (91.5 %).

Six different models analyzed the effect on the number of care interventions proposed. The effect of the study intervention (model 1), age and gender (model 2), number of medicines (model 3), number of episodes (model 4), all independent variables (model 5), and the study intervention together with number of medicines (model 6). The study intervention and the number of medicines showed a significant effect on the number of care interventions proposed. Table 2 shows the effect of models 5 and 6. Looking at the differences between models 5 and 6, AIC and BIC were lower for model 6 and thus considered preferable. According to Raftery [24] a difference of over 10 between the BIC of models 5 and 6 is associated as “very strong” evidence.

**Table 2:** Effect of independent variables on number of care interventions proposed (n = 433 patients)

	Model 5			Model 6		
	Estimate	SE	*p* value^c^	Estimate	SE	*p* value^c^
Study intervention	1.657	0.317	<0.001*	1.662	0.317	<0.001*
Age	0.005	0.013	0.723			
Gender	−0.158	0.142	0.265			
# Medicines^a^	0.045	0.023	0.049*	0.055	0.025	0.030*
# Episodes^b^	0.018	0.012	0.121			
Model fit information						
AIC	715.8			714.2		
BIC	748.4			734.6		

SE, standard error; #, number; AIC, Akaike information criteria; BIC, Bayesian information criteria

* Sign. (*p* value < 0.05)

^a^ATC-coded [22]

^b^ICPC-coded [23]

^c^Multilevel analysis

Table 3 shows the effect of the study intervention on cardiovascular risk factors and safety parameters. Intervention patients had a significantly decreased diastolic blood pressure after 1-year follow-up (79.8–76.8 mmHg; *p* = 0.008). HDL-cholesterol showed a small but significant increase in two groups (intervention patients with intervention: 1.29–1.37 mmol/L; *p* = 0.021; intervention patients without intervention: 1.26–1.37 mmol/L; *p* = 0.039). LDL-cholesterol showed a small but significant decrease in the control group (2.61–2.58 mmol/L; *p* = 0.032). Other parameters showed no significant effect.

**Table 3:** Clinical and laboratory parameters (mean) before study intervention (t = 0) and after 1-year follow-up (t = 1)

	Intervention patients with intervention			Intervention patients without intervention			Control		
	t = 0	t = 1	*p* value^a^	t = 0	t = 1	*p* value^a^	t = 0	t = 1	*p* value^a^
Cardiovascular risk assessment									
BPsystolic (mmHg)	143.7	142.3	0.502	139.0	144.6	0.105	144.3	141.5	0.091
BPdiastolic (mmHg)	79.8	76.8	0.008*	79.5	81.6	0.242	77.6	75.9	0.052
Serum LDL-cholesterol (mmol/L)	2.72	2.63	0.337	2.98	2.67	0.740	2.61	2.58	0.032*
Serum HDL-cholesterol (mmol/L)	1.29	1.37	0.021*	1.26	1.37	0.039*	1.30	1.36	0.074
Serum cholesterol (mmol/L)	4.77	4.77	0.976	4.96	4.75	0.986	4.61	4.61	0.193
BMI (kg/m^2^)	29.8	29.5	0.371	29.9	29.6	0.089	29.9	29.7	0.491
Blood glucose (mmol/L)	6.42	6.56	0.460	6.81	6.51	0.853	6.70	6.72	0.365
HbA1c (mmol/mol)	6.25	6.35	0.213	6.40	6.47	0.226	6.54	6.47	0.582
Safety									
Creatinine clearance (mL/min)	65.1	65.4	0.933	62.1	64.7	0.516	69.1	67.8	0.624
Serum sodium (mmol/L)	139.7	139.9	0.575	138.7	139.5	0.282	139.0	139.4	0.244
Serum potassium (mmol/L)	4.2	4.2	0.601	4.3	4.3	0.081	4.2	4.1	0.681

BP, blood pressure; BMI, body mass index

* Sign. (*p* value < 0.05)

^a^Multilevel analysis

## Discussion

A CMR followed by a PCP resolves almost 50 % of potential harmful DPRs and PCIs (1.0/patient). Differences in percentages of care interventions implemented were observed in different categories. Higher percentages were found for “easy to implement” interventions like stopping a medicine or adjusting a dosage. Interventions from categories taking more time, for example, “additional drug therapy required”, were implemented less frequently. Per patient, an average of 2.2 potential harmful DRPs and PCIs were formulated in the PCP. This is less compared to other studies. In a similar study, 3.5 DRPs and PCIs per patient were found [12], and, after a MR in patients using an automated drug-dispensing system, even a mean of 8.6 potential DRPs per patient was observed [9]. A reason for the lower number of DRPs and PCIs in our study could be the fact that patients seemed well monitored—looking at the initial clinical and laboratory values (Table 3)—and this should be seen in the context of the very low incidence of proposed care interventions in the control group (0.2/patient).

Furthermore, less skill and experience in performing a CMR on the part of primary healthcare providers could have influenced our findings. It is important to have a patient interview as part of the MR process in order to define DRPs [4, 7]. For this reason, pharmacists received a 1-day training course in communication skills and additional written information about how to perform a CMR. More intensive training and experience might help pharmacists perform a CMR better and so define more DRPs. Another reason could be a selection bias, because community pharmacists who had a good relationship with their GPs were recruited, which could have had a positive effect on the quality of medication therapy management. In addition, our study was conducted in regular pharmacies and thereby could reflect regular daily practice more than in studies entailing an extensive training course for pharmacists [11, 12] or with MRs performed by independent pharmacists with several years of experience in performing MRs [9].

Data on interventions performed on control-group patients were collected retrospectively and included only those care interventions where the pharmaceutical care problem was actively documented as a potential DRP and/or CI. A change in medication without a specific documented reason was not included in our data. Therefore, these data might underestimate the number of care interventions for control-group patients and specifically proposed interventions that were not implemented. In daily practice, we expect that, when a pharmacist proposes an intervention to a patient’s GP, not all the proposed interventions will be actively documented in the patient file, especially when the GP and/or patient does not agree with the intervention. Many medication changes occur during a patient’s treatment, but the reason for a care intervention is not always documented.

The second objective of our study, related to the patient efficacy outcome in terms of improvement in cardiovascular risk factors, showed small biochemical changes. It should be noted that baseline clinical and laboratory parameters already showed acceptable values, so the room for improvement was small. Biochemical changes did occur, including changes in HDL- and LDL-cholesterol, but differences were small and not considered clinically relevant. We performed this study in primary care settings with a certain level of cooperation between pharmacists and GPs, who more commonly discuss patient outcomes on a regular basis. In future studies the effects of level of cooperation between pharmacists and GPs on patient outcomes would be of interest. One pharmacist voluntarily registered the total time spent per patient, indicating an average of 145 min per patient with the GP spending an average of 30 min per patient. Patient consultation took an average of 30–60 min per patient.

The study protocol had some main requirements about performing the intervention and data collection (involvement of pharmacist, GP, and patient and the use of the W-PCP application), but organizational matters were not described in detail. It was our intention to allow the practice setting to develop this, as was considered appropriate. Each site could decide how to plan patient consultations and discussions of care plans by pharmacist and GP. The study sites were visited regularly to monitor the progress and quality of the study. Main reason for this approach was to have participating healthcare providers (partly) involved in the implementation of the study and thereby more motivated to perform it. A second reason for this approach was to include the intervention in their daily routine, hoping the MRs would be continued after completion of the study. However, since the MRs took up a lot of time and reimbursement was not available outside the study setting, none of the study sites did continue with the MRs after the study was finished. Thus, reimbursement of these services is essential in order to implement CMRs and PCPs in daily practice. Moreover, tools need to be developed to document interventions and to monitor follow-up, which are easy to implement. These problems related to time, organization, and funding should be seen in the context of the small benefit obtained in terms of cardiovascular risk factors and safety parameters. We question therefore whether this intervention is actually necessary for all patients who fit our inclusion criteria. It might be more suitable for more complex patients with multiple potential DRPs at baseline. Age and number of medicines used are not enough to define patients suitable for a CMR. Risk stratification might be necessary to decide which patients benefit from a CMR and which patients might benefit sufficiently from a MR on a lower, more customary level. Dutch pharmacies all have access to an extensive computer system where automatic checks are performed on drug–drug interactions, contraindications, and duplicate medications. Figure 2 shows that these care issues did not occur in our study population. Instead, they are all dealt with during the daily dispensing of medicines, based on the principle that the pharmacist has approved all dispensed medications after consulting the patient or prescriber.

### Limitations

A lower number of patients than needed for sufficient power in this study were recruited (512 vs. 800). It was hard for pharmacists to motivate GPs to participate. More than 25 pharmacists responded to our letter, but only eight actually decided to participate. Furthermore, during the study period it was hard for pharmacists and GPs to perform CMRs for all intervention patients due to limited time. As a consequence, 70 patients (28 %) from the intervention group had not received any part of the intervention by the end of the study period.

Patients from the control group could have known that they were allocated to the control group when they were not invited to make an appointment with their pharmacist which might have had an impact on their behavior.

We chose a follow-up of 1-year in order for healthcare providers to have sufficient time to implement proposed interventions. When multiple interventions are proposed, healthcare providers may start implementing the most important one. We did not register the time it took to implement each individual intervention. We analyzed how many interventions were implemented after 1-year follow-up.

In the design of our W-PCP the decision was made to have “free text fields” for the care plans instead of pre-defined codes. The main reasons for this were not to bother healthcare providers too much with additional information to document, and to prevent differences in interpretation and coding by different healthcare providers. Therefore, information from the care plans was coded after the study period by the researchers. A total of thirty-six potential harmful DRPs and PCIs were not described properly and could not be coded. This might have created bias.

## Conclusion

Healthcare providers sharing information electronically are capable of performing integrated care for their patients by conducting CMRs and developing PCPs. The integrated use of a CMR with a PCP supports detection and decreases DRPs and PCIs. However, its benefit in terms of efficacy and safety parameters is relatively low in a primary, well-regulated, low-risk population. It might have been more efficient in terms of outcomes if a higher-risk target group had been selected.
